# Decreased level of irisin, a skeletal muscle cell-derived myokine, is associated with post-stroke depression in the ischemic stroke population

**DOI:** 10.1186/s12974-018-1177-6

**Published:** 2018-05-02

**Authors:** Wen-Jun Tu, Han-Cheng Qiu, Qiang Liu, Xuemei Li, Ji-Zong Zhao, Xianwei Zeng

**Affiliations:** 1 Institute of Radiation Medicine, China Academy of Medical Science and Peking Union Medical College, Tianjin, China; 2 Department of Neurosurgery, Beijing Tiantan Hospital of Capital Medical University, Beijing, China; 3 Center for Translational Medicine, Institutes of Stroke, Weifang Medical University, Weifang, China; 4 Tianjin, China; 5 Beijing, China

**Keywords:** Irisin, Depression, Acute ischemic stroke, Chinese

## Abstract

**Background:**

Depression is a frequent mood disorder in stroke patient. Our aim was to determine irisin levels in serum and investigate their associations with post-stroke depression (PSD) in a 6-month follow-up study in Chinese patients with first-ever acute ischemic stroke (AIS).

**Methods:**

The subjects were first-ever AIS patients who were hospitalized at three stroke centers during the period from January 2015 to December 2016. Neurological and neuropsychological evaluations were conducted at the 6-month follow-up. Serum irisin concentrations were measured by enzyme-linked immunosorbent assay (ELISA).

**Results:**

During the study period, 1205 patients were included in the analysis. There were 370 patients (30.7%) classified as depression. The depression distribution across the irisin quartiles ranged between 49.8% (first quartile) and 9.9% (fourth quartile). In the patients with depression, serum irisin levels were lower compared with those in patients without depression (*P* < 0.001). In a multivariate model using the first (Q1) quartile of irisin vs. Q2–4 together with the clinical variables, the marker displayed predictive information and increased risk of PSD by 75% (odds ratio [OR] for Q1, 1.75 [95% confidence interval [CI], 1.15–2.65]). In addition, a model containing known risk factors plus irisin compared with a model containing known risk factors without irisin showed a greater discriminatory ability; the area under the curve (AUC) increased from 0.77 to 0.81 (95% CI, 0.76–0.86).

**Conclusions:**

The data suggested that reduced serum levels of irisin were powerful biological markers of risk of developing PSD even after adjustment by variables. Further studies are necessary to confirm this association, which may open the way to the proposal of new therapeutic options.

**Trial registration:**

ChiCTR-OPC-17013501. Retrospectively registered 23 September 2017

## Background

Stroke is a medical emergency caused by interrupted blood supply to the brain that further leads to rapid loss of brain functions. It is the second leading cause of death worldwide and associated with long-term disability [1]. Post-stroke depression (PSD) is a frequent mood disorder in stroke patient. It worsened stroke-related outcomes in the form of greater functional disability and higher mortality [2]. Hadidi et al. [3] suggested that patients with PSD shown far less recovery from functional impairments compared with no depression patients with stroke and were 3.4 times more likely to die during the first 10 years after stroke.

Irisin is a small polypeptide hormone that is cleaved from the fibronectin type III domain containing 5 (FNDC5) [4]. The secreted level of irisin is commonly increased by exercise [5]. The highest basal levels of FNDC5 expression are seen in the brain and heart, with low basal levels in the liver, lung, skeletal muscle, and testis [6]. Irisin might be the master hormone to decide whether energy is released as heat or stored as ATP.

Accumulating evidence has demonstrated that irisin contributes to the regulation of glucose and lipid metabolism in the skeletal muscle and adipose tissue [7, 8]. Besides, irisin was also found to have an essential role in the chronic kidney disease [9], obesity [10], insulin resistance [11], or type 2 diabetes [12]. Zhang et al. [13] observed that physical exercise may signal to the brain to modulate metabolic activity and locomotion via irisin. Furthermore, irisin-encoding gene (FNDC5) variant can change blood pressure in men with type 2 diabetes [14], while irisin improves endothelial function in type 2 diabetes [15] and in a mouse model of obesity [16]. In addition, irisin protects against endothelial injury and ameliorates atherosclerosis in Apo-E knockout mice [17]. Due to the crosstalk between metabolic dysfunction and cardio-cerebrovascular diseases, thus, a role for irisin in the cardio-cerebrovascular system is expected.

Furthermore, irisin plays substantial roles in the pathophysiology of metabolic diseases [18]. Metabolic diseases are associated with depression and depressive symptoms [19, 20]. Moreover, irisin has been found to be not only a myokine but also an adipokine [21]. Evidences suggest that adipokines are involved in depression pathophysiology [22]. Wang and Pan [23] had demonstrated that irisin has a crucial role in inducing antidepressant-like effects in chronic unpredictable stress (CUS) rats by regulating energy metabolism in the prefrontal cortex of the brain.

A previous meta-analysis found that exercise was an evidence-based treatment for depression [24], and this effect has been linked to a PGC-1α-FNDC5/irisin pathway, which is activated by exercise in the hippocampus in mice and induces a neuroprotective gene program [25]. Furthermore, a previous study showed that a low serum irisin level was a predictor of poor early functional outcome in ischemic stroke patients [26]. We hypothesized that irisin serum level is a risk factor for depression among patients with ischemic stroke. The aim of this study is to determine irisin levels in serum and investigate their associations with PSD in a 6-month follow-up study in Chinese patients with first-ever acute ischemic stroke (AIS).

## Methods

### Study population

The subjects were first-ever AIS patients who were hospitalized at three stroke centers (Beijing, Weifang, and Wuhan) during the period from January 2015 to December 2016. AIS was defined according to the World Health Organization Multinational Monitoring of Trends and Determinants in Cardiovascular Disease (WHO-MONICA) criteria and was verified by magnetic resonance imaging (MRI) reports performed within 24 h after admission. Exclusion criteria were as follows: (1) pre-stroke diagnosis of dementia or cognitive impairment, a history of any psychiatric illness, and decreased level of consciousness; (2) malignant tumor, sarcopenia, liver insufficiency and renal insufficiency (creatinine > 1.5 mg/dL), metabolic abnormalities (not included diabetes), severe edema, and autoimmune diseases; (3) used psychotropic drugs prior to stroke onset; (4) without informed consent, lost blood samples, or current medications used that influence serum irisin levels; and (5) death during the follow-up.

The control group consisted of 120 healthy volunteers (sex and age matched) without a history of psychiatric or neurological disorders. The control group also received clinical assessment, and their score on the Hamilton Scale was less than 7. Written informed consent were obtained from all patients, and this study conformed to the principles of the Declaration of Helsinki and was approved by the investigational review board of the Weifang Medical University.

### Clinical variables and follow-up

At baseline, demographic data (age, sex, and body mass index [BMI]) and the following vascular risk factors, hypertension, diabetes mellitus, hypercholesterolemia, smoking, and a family history of ischemic stroke, were collected. Pre-stroke therapy (oral anticoagulants or antiplatelet agents) and acute treatment (IV thrombolysis and/or mechanical thrombectomy) were also recorded. Patients were evaluated with the National Institute of Health Stroke Scale (NIHSS) [27] score at their admission, performed by a stroke neurologist certified. Stroke subtype was classified according to TOAST (Trial of Org 10172 in Acute Stroke Treatment) criteria. Brain imaging (MRI) was done routinely within 24 h after admission. MRI with diffusion-weighted imaging (DWI) was available for some patients. The infarct volume was calculated by using the formula 0.5 × *a* × *b* × *c* (where *a* is the maximal longitudinal diameter, *b* is the maximal transverse diameter perpendicular to *a*, and *c* is the number of 10-mm slices containing infarct) [28].

The end point was psychological evaluation on month 6 after admission. Psychological evaluation was performed by the trained psychologist at 6 months. All patients were interviewed using the structured clinical interview of DSM-IV (SCID-I-R) [29], allowing for a diagnosis of both major and minor depression. Inter-rater reliability was examined in 100 patients, and the kappa was 0.92. The severity of depressive symptoms was measured with the 17-item Hamilton Depression Rating Scale (HAM-D) [30]. We have used the validated version for the Chinese population. Data concerning demographics, marital status, living situation, family history of psychiatric disorders, and drug treatment were also collected by interview. Functional outcome was also obtained according to the Modified Rankin Scale (mRS) score [31]. The evaluation was conducted by a neurologist/psychiatrist who was unaware of the type, size, and location of the index stroke at the time of the investigation and throughout the diagnostic procedure. In addition, specific details about physical activity in some patients (*n* = 223) were assessed by a validated questionnaire adapted from the Canadian Community Health Survey [32].

### Blood collection and laboratory test

All blood samples were collected on the first day of admission under fasting state. Blood samples were centrifuged at 1000×*g* for 12 min, and the serum was separated and stored at − 80 °C until the time of assay. Biochemical measurements were done using standard laboratory methods. The levels of serum total cholesterol (TC), triglycerides (TG), high-sensitivity C-reactive protein(Hs-CRP), glucose, homocysteine (HCY), and high-density lipoprotein cholesterol (HDL-C) were measured by enzymatic assays, and fasting glucose levels were measured using the hexokinase method (Autoanalyzer Model 7600 II; Hitachi, Tokyo, Japan). Insulin resistance (IR) was estimated from fasting serum measurements using the homeostasis model assessment of insulin resistance (HOMA-IR): insulin (μIU/mL) × glucose (mmol/L)/22.5. Serum irisin concentrations were measured in duplicate by using the enzyme-linked immunosorbent assay (ELISA) kits (Code No. SK00170-09; Aviscera Biosciences, Santa Clara, CA, USA), in accordance with the manufacturer’s instructions. The sensitivity of the assay was 1.0 ng/mL, and the linear range of the standard was 1–500 ng/mL. The intra-and inter-assay coefficients of variation (CV) were 3.3–5.0% and 4.3–6.8%, respectively. Serum interleukin-6 (IL-6) was determined by ELISA (Human IL-6 Quantikine ELISA Kit [Code No. D6050], R&D Systems, Inc., Minneapolis, MN, USA). The sensitivity of the assay was 0.7 pg/mL, and the linear range of the standard was 3–300 pg/mL. The intra- and inter-assay CV were 1.7–4.4% and 3.8–6.4%, respectively. Serum levels of serotonin were quantitatively determined via direct competitive radioimmunoassay (RIA) with the separation of bound/free ligands using an IgG antibody (ALPCO Diagnostics, Salem, NH 03079, USA). The manufacturer’s instructions were followed. The results were reported in nanograms per milliliter. The analytical sensitivity of this serotonin RIA is 0.3 ng/mL with the CV for the intra- and inter-assay reproducibility as 2.5–4.7 and 3.6–5.8%, respectively. For all measurements, the levels that were not detectable were considered to have a value equal to the lower limit of detection of the assay. Determinations were performed in an independent laboratory blinded to clinical and neuroimaging data.

### Statistical analysis

Results are expressed as percentages for categorical variables and as means (standard deviation, S.D.) or medians (quartiles) for the continuous variables, depending on the normal or non-normal distribution of data. Proportions were compared using the *χ*^2^ test, and Student’s *t* test and analysis of variance (ANOVA) were employed for the normally distributed variables, while the Mann–Whitney *U* test was employed for the asymmetrically distributed variables. Spearman’s rank correlation was used for bivariate correlations.

The relationship between serum levels of irisin and PSD were evaluated using univariate and multivariate regression analyses. We used crude models and multivariate models adjusted for all significant predictors and report odds ratios (ORs). For multivariate analysis, categorical variables included age, sex, BMI, stroke etiology, the NIHSS score, infarct volume, pre-stroke and acute treatment, vascular risk factors, mRS at follow-up, family history of psychiatric disorders, lesion location, living with offspring, widowhood, HOMA-IR, blood levels of glucose, Hs-CRP, HCY, IL-6, TG, TC, HDL, LDL, and serotonin and irisin quartiles. For a more detailed exploration of the irisin and PSD, we also used multivariate analysis models to estimate adjusted OR and 95% CIs of PSD for irisin quartiles (with highest irisin quartile as reference). In addition, serum irisin level was also dichotomized according to irisin quartiles, and the grouping of quartiles 2–4 was defined as normal while the grouping of quartile 1 as low.

Second, receiver operating characteristic (ROC) curves was used to test the overall predict accuracy of irisin and other markers to diagnose PSD, and results were reported as area under the curve (AUC). Integrated discrimination improvement (IDI) and net reclassification improvement (NRI) indices were calculated to determine the clinical utility of the addition of irisin to established risk factors and the ability of irisin to improve PSD prediction [33].

Finally, the sensitivity analysis was performed to test the robustness of the results, and only those patients with information about physical activity were included. In multivariate logistic regression analysis, we calculated the OR of irisin level to predict PSD after adjusted physical activity and other risk factors. All statistical analyses were performed with SPSS for Windows, version 21.0 (SPSS Inc., Chicago, IL, USA), the ROCR package (version 1.0-2), and the GraphPad Prism 5.0. Statistical significance was defined as *P* < 0.05.

## Results

### Baseline characteristics of study samples

**Fig. 1:**
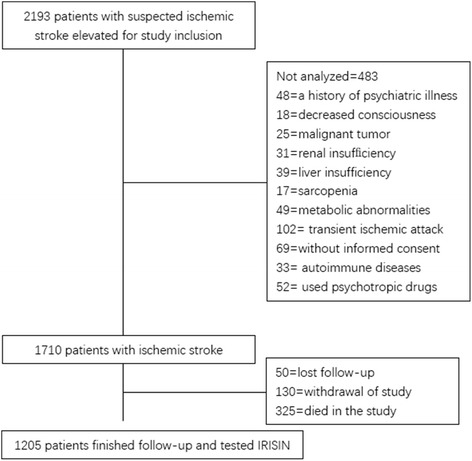
Study profile/flow sheet of the study

The study cohort consisted of 1710 patients at stroke admission. By the time of 6-month follow-up, 325 had passed away, 130 declined the invitation to participate, and 50 had lost follow-up, leaving 1205 individuals (Fig. 1). However, these included patients were similar in terms of baseline characteristics [age (*P* = 0.18), sex (*P* = 0.69), and BMI (*P* = 0.42)] compared to the overall cohort. Overall, the median age was 65 (IQR, 56–75), and 640 (53.1%) were male. The irisin levels were obtained in stroke patients with a median value of 95.9 ng/mL (IQR, 71.1–144.0 ng/mL), which was significantly lower than the levels in controls (137.5; IQR, 100.4–171.5 ng/mL). The baseline characteristics of the 1205 stroke patients with and without PSD were described in Table 1.

**Table 1:** Baseline clinical characteristics in patients with and without post-stroke major depression at 6 months

	No depression	Depression	*P*
*N*	835	370	–
Age (years), median (IQR)	64 (56–75)	69 (57–78)	0.001
Male, *n* (%)	430 (51.5)	210 (56.8)	0.450
BMI (kg/m^2^), median (IQR)	26.8 (24.2–28.7)	27.2 (24.3–28.8)	0.312
Stroke severity, median NIHSS score (IQR)	6 (3–9)	11 (6–16)	0.005
Lesion volumes (mL), median (IQR)	32 (7–61)	48 (14–75)	0.009
mRS at 3 months, median (IQR)	1 (0–2)	1 (1–2)	0.252
Vascular risk factors (%)			
Hypertension	67.1	70.2	0.683
Diabetes mellitus	34.1	38.3	0.597
Coronary heart disease	31.1	34.0	0.706
Family history for stroke	21.0	25.5	0.503
Current smoking	22.2	23.4	0.856
Pre-stroke treatment (%)			
Antiplatelet agents	31.7	34.0	0.765
Anticoagulants	21.0	23.4	0.718
Endovascular/surgical revascularization (%)			
Family history of psychiatric disorders (%)	10.2	27.7	0.002
Widowhood (%)	9.0	25.5	0.003
Living with offspring (%)	10.2	27.7	0.002
Stroke etiology (%)			0.623
Atherothrombotic	32.9	29.8	
Cardioembolic	28.7	25.5	
Lacunar	16.8	23.4	
Unknown	21.6	21.3	
Lesion location (%)			0.371
Frontal	26.9	21.3	
Parietal	14.4	19.1	
Basal ganglia	20.4	21.3	
Posterior fossa	17.4	21.3	
Other	21.0	17.0	
Laboratory findings, median (IQR)			
Glucose (mmol L^−1^)	6.18 (5.54–6.75)	6.22 (5.58–6.85)	0.206
Hs-CRP (mg dL^−1^)	0.48 (0.24–0.69)	0.75 (0.46–1.23)	0.009
HCY (mmol L^−1^)	13.8 (9.6–16.5)	17.2 (12.1–21.6)	0.018
TC (mmol L^−1^)	4.86 (4.32–5.36)	4.90 (4.32–5.41)	0.428
TG (mmol L^−1^)	1.89 (1.43–2.21)	1.88 (1.40–2.26)	0.501
HDL-C (mmol L^−1^)	1.16 (1.03–1.24)	1.18 (1.05–1.29)	0.332
LDL-C (mmol L^−1^)	2.48 (2.11–2.76)	2.40 (2.04–2.83)	0.242
IL-6 (pg mL^−1^)	101.2 (76.5–138.6)	132.2 (102.3–164.8)	0.003
HOMA-IR	2.13 (1.52–3.04)	2.20 (1.62–3.11)	0.151
Serotonin (ng mL^−1^)	1.65 (0.39–3.83)	1.18 (0.35–2.96)	< 0.001
Irisin (ng mL^−1^)	120.3 (77.6–164.8)	74.2 (60.4–108.4)	< 0.001

Results are expressed as percentages for categorical variables and as medians (quartiles) for the continuous variables

*NIHSS* National Institutes of Health Stroke Scale, *mRS* Modified Rankin Scale, *S.D.* standard deviation, *IQR* interquartile range, *Hs-CRP* high-sensitivity C-reactive protein, *HCY* homocysteine, *BMI* body mass index, *HDL-C* high-density lipoprotein cholesterol, *LDL-C* low-density lipoprotein cholesterol, *TG* triglyceride, *TC* total cholesterol, *HOMA-IR* homeostatic model of assessment insulin resistance

### Main findings

**Fig. 2:**
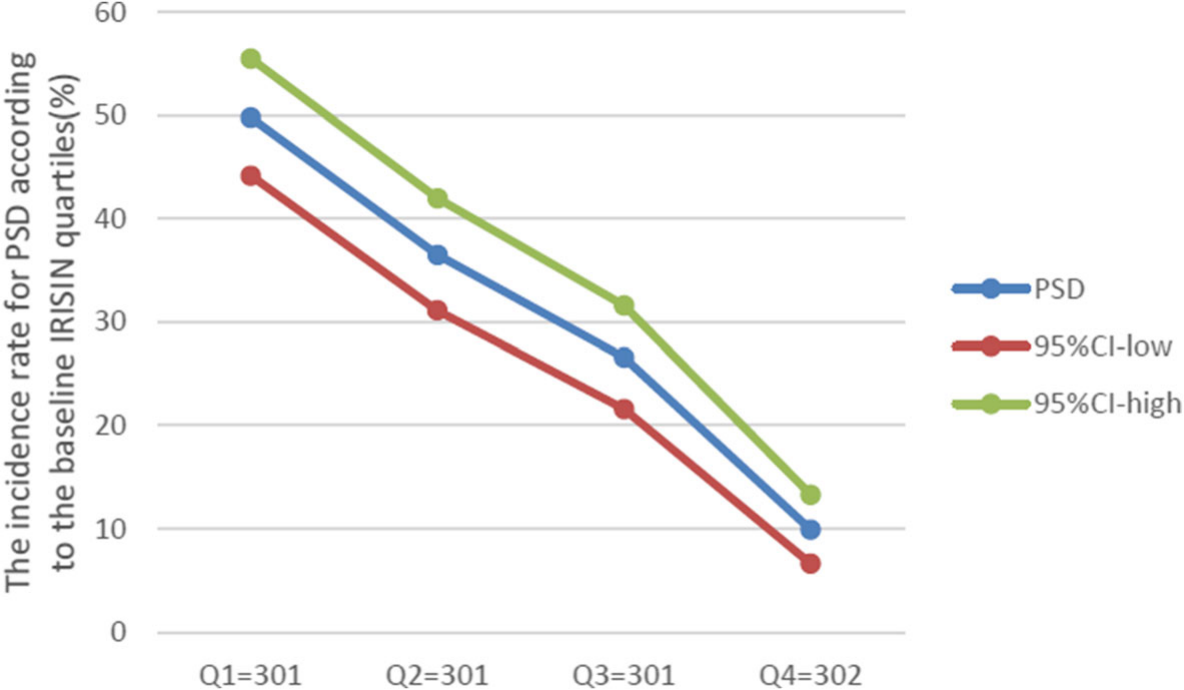
The incidence for PSD according to the baseline irisin quartiles. Serum levels of irisin in quartile 1 (< 71.1 ng/mL), quartile 2 (71.1–95.9 ng/mL), quartile 3 (96.0–144.0 ng/mL), and quartile 4 (> 144.0 ng/mL). PSD post-stroke depression

During the 6-month follow-up period, all surviving patients were examined regarding depression. There were 370 patients (30.7%, 95% CI 28.1–33.3%) classified as depression. The depression distribution across the irisin quartiles ranged between 49.8% (first quartile) to 9.9% (fourth quartile) (Fig. 2). Patients with depression were older and more frequently were widowhood, living with offspring, higher initial stroke severity, larger infarct volume, higher serum levels of Hs-CRP and HCY, and lower levels of serotonin (Table 1).

Serum levels of irisin decreased with increasing severity of stroke as defined by the NIHSS score. There was a negative correlation between levels of irisin and the NIHSS (*r* [spearman] = − 0.343, *P* < 0.001). There were modest correlations between levels of irisin and Hs-CRP (*r* = − 0.232, *P* < 0.001), IL-6 (*r* = − 0.287, *P* < 0.001), HCY (*r* = − 0.269, *P* < 0.001), serotonin (*r* = 0.159, *P* = 0.12), and HDL (*r* = 0.188, *P* = 0.001). In addition, there is a negative correlation between levels of irisin and BMI (*r* = − 0.273, *P* < 0.001). There was no correlation between levels of irisin and sex (*P* = 0.24) and age (*P* = 0.11). Furthermore, there was a modest negative correction between irisin and HAM-D score (*r* = − 0.211, *P* < 0.001). There was still a significant negative correction between irisin serum levels and HAM-D score, using ordered logistic regression after multivariate adjustment for possible confounders (*P* = 0.003).

**Fig. 3:**
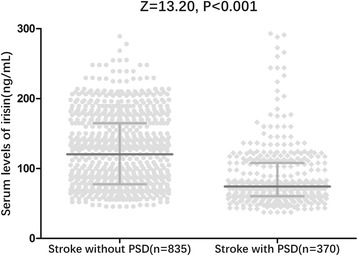
Distribution of serum levels of irisin according to stroke patients with and without PSD. All data are medians and interquartile ranges (IQR); *P* values refer to Mann–Whitney *U* tests for differences between groups. PSD post-stroke depression

In the 370 patients with depression, serum irisin levels were lower compared with those in patients without depression [74.2 (IQR, 60.4–108.4) ng/mL vs. 120.3 (IQR, 77.6–164.8) ng/mL; *P* < 0.001; Fig. 3]. In univariate logistic regression analysis, we calculated the odds ratio (OR) of irisin level to predict PSD as compared with the NIHSS score and other risk factors. With an unadjusted OR of 0.981 (95% CI, 0.974–0.989; *P* < 0.001), irisin had a strong association with PSD. After adjusting for all other significant outcome predictors, irisin remained an independent PSD predictor with an adjusted OR of 0.989 (95% CI, 0.985–0.995). In multivariate models comparing the first (Q1), second (Q2), and third (Q3) quartiles against the fourth (Q4) quartile of irisin (Table 2), levels of irisin in Q1, Q2, and Q3 were associated with PSD and increased risk of PSD by 385% (OR = 4.85; 95% CI, 3.30–7.92), 179% (OR, 2.79; 95% CI, 1.82–4.37), and 82% (1.82; 1.13–2.92). The independent association of irisin with PSD was confirmed using the likelihood ratio test (*P* = 0.002). In a multivariate model using the Q1 of irisin vs. Q2–4 together with the clinical variables, the marker displayed prognostic information and increased risk of PSD by 75% (OR for Q1, 1.75 [95% CI, 1.15–2.65]).

**Table 2:** Odds ratios for PSD according to irisin quartiles at admission

Irisin quartiles^ǂ^	PSD, *N*	Unadjusted OR (95% CI)^ξ^	Adjusted OR (95% CI)*^ξ^
Q1, *N* = 301	150	9.01 (5.80–13.98)	4.85 (3.30–7.92)
Q2, *N* = 301	110	4.96 (3.19–7.73)	2.79 (1.82–4.37)
Q3, *N* = 301	80	3.28 (2.08–5.18)	1.82 (1.13–2.92)
Q4, *N* = 302	30	References	References

*OR* odds ratio, *CI* confidence interval, *PSD* post-stroke depression, *NIHSS* National Institutes of Health Stroke Scale, *mRS* Modified Rankin Scale, *Hs-CRP* high-sensitivity C-reactive protein, *HCY* homocysteine, *BMI* body mass index, *HDL-C* high-density lipoprotein cholesterol, *LDL-C* low-density lipoprotein cholesterol, *TG* triglyceride, *TC* total cholesterol, *HOMA-IR* homeostatic model of assessment insulin resistance, *IL-6* interleukin-6

^ǂ^Serum levels of irisin in quartile 1 (< 71.1 ng/mL), quartile 2 (71.1–95.9 ng/mL), quartile 3 (96.0–144.0 ng/mL), and quartile 4 (> 144.0 ng/mL)

^*^Adjusted for age, sex, BMI, stroke etiology, the NIHSS score, infarct volume, pre-stroke and acute treatment, vascular risk factors, mRS at follow-up, family history of psychiatric disorders, lesion location, living with offspring, widowhood, HOMA-IR, blood levels of glucose, Hs-CRP, HCY, IL-6, TG, TC, HDL, LDL, and serotonin and irisin quartiles

^ξ^*P* value for the trend < 0.001

**Fig. 4:**
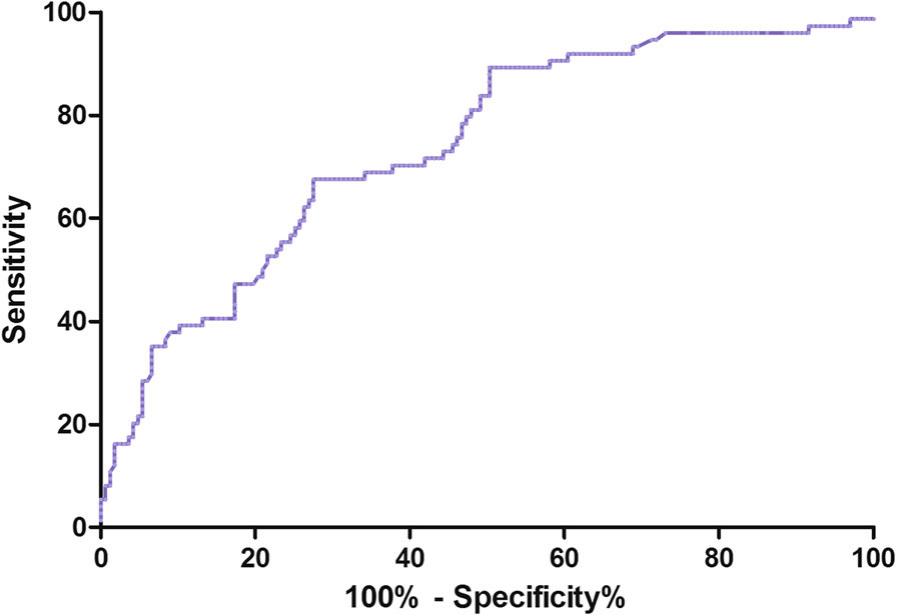
Receiver operator characteristic curve demonstrating sensitivity as a function of 1-specificity for predicting the PSD within 6 months based on the serum irisin levels at admission. PSD post-stroke depression

Using ROC curves, irisin levels > 85 ng/mL at admission predicted the development of depression at 3 months with the highest sensitivity and specificity [67.6 and 70.7%, respectively; area under the curve (AUC) = 0.74; 95% CI, 0.67–0.81] (Fig. 4). With an AUC of 0.74, irisin showed a significantly greater discriminatory ability to predict PSD as compared with Hs-CRP (AUC, 0.61; 95% CI, 0.56–0.67; *P* < 0.001), HCY (AUC, 0.66; 95% CI, 0.59–0.72; *P* = 0.002), age (AUC, 0.59; 95% CI, 0.53–0.65; *P* < 0.001), NHISS score (AUC, 0.60; 95% CI, 0.55–0.66; *P* < 0.001), and serotonin(0.69, 0.59; 95% CI, 0.63–0.76; *P* = 0.009).

In addition, a model containing known risk factors plus irisin compared with a model containing known risk factors without irisin showed a greater discriminatory ability; the area under the curve (AUC) increased from 0.77 to 0.81 (95% confidence interval [CI], 0.76–0.86). A significant difference in the AUC between the clinical variables alone and the addition of irisin level was observed (difference, 0.04 [95% CI, 0.03–0.05]; *P* = 0.02) (Table 3). The NRI statistic showed that the addition of irisin to established risk factors significantly increased the correct reclassification of depressive patients and non-depressive patients(*P* = 0.003). The IDI statistic also found that the irisin level significantly increased discrimination between depressive patients and non-depressive patients (*P* = 0.01).

**Table 3:** Serum irisin concentrations at admission prediction of PSD with AUROC

PSD	AUROC					
	Irisin	Risk factors^※^	Risk factors with IRISIN^※^	Incremental area (*P*)^**‡**^	NRI (*P*)	IDI (*P*)
At admission	0.74	0.77	0.81	0.04 (0.02)	0.13 (0.003)	0.06 (0.01)

*PSD* post-stroke depression, *NIHSS* National Institutes of Health Stroke Scale, *mRS* Modified Rankin Scale, *BMI* body mass index, *Hs-CRP* high-sensitivity C-reactive protein, *HCY* homocysteine, *HDL-C* high-density lipoprotein cholesterol, *LDL-C* low-density lipoprotein cholesterol, *TG* triglyceride, *TC* total cholesterol, *HOMA-IR* homeostatic model of assessment insulin resistance, IL-6 interleukin-6

^※^Established risk factors including age, sex, BMI, stroke etiology, the NIHSS score, infarct volume, pre-stroke and acute treatment, vascular risk factors, mRS at follow-up, family history of psychiatric disorders, lesion location, living with offspring, widowhood, HOMA-IR, blood levels of glucose, Hs-CRP, HCY, IL-6, TG, TC, HDL, LDL, and serotonin

^‡^Comparison of AUROCs: established risk factors without irisin levels vs. established risk factors with irisin levels

### Subgroup analysis

In the subgroup of patients (*n* = 223) in whom physical activity evaluations were performed, 69 (30.9%) patients were defined as depression patients. Physical activity in stroke patients with depression were lower compared with those in patients without depression [1.0 (IQR, 0.7–1.5) kcal/kg day vs. 1.3 (IQR, 0.8–1.7) kcal/kg day; *P* = 0.001]. There was a positive correlation between levels of irisin and the physical activity (*r* [spearman] = 0.173, *P* = 0.015). Physical activity in stroke patients with low level of irisin (Q1) were lower compared with those in patients with normal irisin (Q2–4) [0.8 (IQR, 0.5–1.2) kcal/kg day vs. 1.5 (IQR, 1.0–1.9) kcal/kg day; *P* < 0.001]. In multivariate logistic regression analysis, we calculated the OR of irisin level to predict PSD after adjusted physical activity and other risk factors. Irisin was still an independent PSD predictor with an OR of 0.992 (95% CI, 0.985–0.997; *P* = 0.003) after adjustment for both physical activity and other risk factors. Similarly, in a multivariate model using the low (Q1) of irisin vs. normal (Q2–4) together with physical activity and other risk factors, the marker displayed prognostic information and increased risk of PSD by 63% (OR for Q1, 1.63 [95% CI, 1.12–2.57]).

## Discussion

A review identified potential biopsychosocial variables associated with the relationship between obesity and depression [34]. This is the first study to date to assess concentration of irisin, a risk of obesity [35], at admission in relation to the development of PSD and to investigate its clinical utility in Chinese stroke patients. The findings of this study were as follows: (1) serum irisin levels in patients with depression were lower compared with those in patients without depression; (2) reduced serum levels of irisin were powerful biological markers of risk of developing PSD even after adjustment by variables, and thus, it may be used as a future therapeutic target in patients with ischemic stroke; and (3) irisin showed a significantly greater discriminatory ability to predict PSD as compared with other biomarkers, such as Hs-CRP, HCY, age, and serotonin.

Serotonin is strongly associated with the etiology of major depressive disorder and has been suggested as a key contributor to the regulation of mood and anxiety [36]. A previous study found that serotonin deficiency may be one of the factors leading to depression following stroke [37]. In this study, we also found that low levels of serotonin were useful biomarkers to predict PSD. However, the predictive efficiency was lower than irisin (*P* = 0.009). In addition, the predicted trends of irisin for PSD had not been changed, even after adjusted for serotonin in the multivariate analysis.

In this study, we found that serum levels of irisin in stroke patients were lower than in controls. Consistent with our results, Li et al. [38] reported that the plasma irisin concentration and intramuscular FNDC5 protein expression decreased after ischemic stroke. In this same study, the author confirmed that plasma irisin levels were negatively associated with brain infarct volume, the neurological deficit score, and plasma TNF-α and plasma IL-6 concentrations [38]. Similarly, our study suggested that irisin levels were negatively associated with infarct volume, the neurological deficit score, serum levels of CRP and IL-6. A previous study have shown that irisin levels were lower in myocardial infarction (MI) and coronary atherosclerosis diseases (CAD) implying that their production may depend on myocardial blood supply [39], while another study suggested that serum irisin level was an independent predictor of coronary artery severity in patients with stable coronary artery disease [40].

Although previous results have provided evidence that irisin may mediate some of the positive effects of training on body weight, insulin sensitivity, and glucose homeostasis, studies in humans have led to mixed results. Thus, some studies have reported a positive correlation between BMI, IR and circulating irisin levels [41, 42], or muscle FNDC5 mRNA expression [4, 12], while other studies have reported a negative correlation between BMI and circulating irisin levels [10, 43]. In this study, there is a negative correlation between BMI, IR, and circulating irisin levels.

The mechanism by which irisin mediates the depressive effect on stroke is still unknown. A possible explanation is interplay with inflammation markers. Inflammation may represent a common mechanism of disease has been extended to include neuropsychiatric disorders including major depression [44]. In addition, a study provides evidence of an association between irisin and homocysteine, which may be due to nicotinamide metabolism [45]. Cheng et al. suggested that that elevated serum levels of Hs-CRP and HCY were associated with the risk of developing PSD [46]. However, in this study, irisin remained significantly associated with PSD even after adjustment for Hs-CRP or IL-6, suggesting that the effect of irisin on PSD was independent of inflammation.

Another possible explanation is interplay with other myokines, adipocytokines, or classical cytokines. Shan et al. [47] demonstrated an important interplay between irisin and myostatin, another myokine. A study suggested that the lowered adiponectin levels in depression are depression-specific and not explained by conventional low adiponectin-related factors [48]. Third, lower levels of irisin are independently associated with endothelial dysfunction. Endothelial dysfunction is associated with a greater depressive symptom score in a general elderly population [49]. Moreover, coronary endothelial dysfunction is associated with an increased risk of cerebrovascular events. Irisin alleviates endothelial dysfunction in type 2 diabetes partially via reducing oxidative/nitrative stresses through inhibiting signaling pathways implicating PKC-β/NADPH oxidase and NF-κB/iNOS [15]. A study concludes that acute administration of irisin lowers blood pressure of SHRs by amelioration of endothelial dysfunction of the mesenteric artery through the AMPK-Akt-eNOS-NO signaling pathway [50]. Fourth, a previous study indicated that irisin serves as a novel approach to eliciting cardioprotection, which is associated with the improvement of mitochondrial function [2]. Della et al. [51] reported that complexes I, III, and IV of the mitochondrial respiratory chain were inhibited by chronic mild stress in the cerebral cortex and cerebellum, contributing to depressive-like behaviors in rats. Lastly, since the discovery of irisin, its levels have been associated with both cholesterol and steroid hormones, such as estradiol [41], which leads us to believe that it might be involved in the process of depression [52]. Hence, further studies should explore the molecular mechanism to elucidate direct irisin effects.

### Strengths and limitations

The strengths of our study include the fact that it is a prospective multiethnic study with a relatively larger sample, making the results robust and generalizable. Furthermore, we collected data on a wide range of potentially confounding risk factors, allowing us to estimate the independent effect of irisin on PSD. Lastly, we chose a different strategy using the fourth quartiles, because we have found this strategy less sensitive to other factors that might influence the relatively low concentrations.

The following limitations of our study must be taken into account. First, although the data suggested an inverse correlation of irisin with post-stroke depression, investigation of cause–effect and mechanisms might be a more interesting question. These studies help to analyze whether the change of circulating irisin was pathogenically involved in the development of PSD or just a mark. However, the cross-sectional nature of the study precludes us to draw any conclusion on the role of irisin in the development of PSD, and therefore, no conclusion regarding cause–effect and mechanism relationships can be made. Therefore, the impact of irisin on future depression outcomes should be clarified in the future study. Second, irisin is usually measured by ELISA, but the quantification varies greatly between the kits. These differences probably come from the variety in the irisin epitopes being targeted for measurement by the manufacturing companies. Interestingly, two recent reports challenged the putative presence of irisin in human plasma [53]. Third, in the present study, there was no information in irisin levels according to regular exercise. However, concentrations of irisin increased significantly after endurance exercise training in both mice and humans [54]. Fourth, the dead patients had been excluded, who might suffer from depression. This approach may underestimate the incidence of depression. Fifth, PSD is a progressive complication; serum irisin levels in follow-up are valuable. However, in this study, only blood samples at admission were collected. Without serial measurement of the circulating irisin, this study yielded no data regarding when and how long biomarkers were reduced in these patients. Additionally, it should be investigated whether serial irisin testing further improves the risk stratification of PSD patients. Finally, all participants to the present study were Chinese Han, and whether these observations can also be extended to other ethnic groups with different body composition remains to be determined.

## Conclusions

The present study suggested that reduced serum levels of irisin were powerful biological markers of risk of developing PSD even after adjustment by variables. Further studies are necessary to confirm this association, which may open the way to the proposal of new therapeutic options.

## Abbreviations

AIS: Acute ischemic stroke
AUC: Area under the curve
BMI: Body mass index
CUS: Chronic unpredictable stress
CV: Coefficients of variation
DWI: Diffusion-weighted imaging
ELISA: Enzyme-linked immunosorbent assay
FNDC5: Fibronectin type III domain containing 5
HAM-D: Hamilton depression rating scale
HCY: Homocysteine
HDL-C: High-density lipoprotein cholesterol
HS-CRP: High-sensitivity C-reactive protein
IDI: Integrated discrimination improvement
IR: Insulin resistance
MRI: Magnetic resonance imaging
mRS: Modified Rankin Scale
NIHSS: National Institute of Health Stroke Scale
NRI: Net reclassification improvement
OR: Odds ratios
PSD: Post-stroke depression
ROC: Receiver operating characteristic curves
TC: Total cholesterol
TG: Triglycerides
TOAST: Trial of Org 10172 in Acute Stroke Treatment
